# DuoStim Shows Comparable Efficacy but Better Efficiency than Two Conventional Stimulations in Poor/Suboptimal Responders Undergoing Vitrified Oocyte Accumulation for PGT-A

**DOI:** 10.3390/life15060899

**Published:** 2025-05-31

**Authors:** Stefano Canosa, Alberto Revelli, Danilo Cimadomo, Alberto Vaiarelli, Gianluca Gennarelli, Daniela Guidetti, Andrea Roberto Carosso, Laura Rienzi, Filippo Maria Ubaldi, Francesca Bongioanni

**Affiliations:** 1 IVIRMA Global Research Alliance, Livet, Via Tiziano Vecellio 3, 10126 Turin, Italy; aerre99@yahoo.com (A.R.); gennarelligl@gmail.com (G.G.); daniela.guidetti@tin.it (D.G.); francesca.bongioanni@generapma.it (F.B.); 2 Gynecology and Obstetrics 2U, Department of Surgical Sciences, S. Anna Hospital, University of Turin, 10126 Turin, Italy; 3 IVIRMA Global Research Alliance, Genera, Clinica Valle Giulia, 00197 Rome, Italy; danilo.cimadomo@ivirma.com (D.C.); albertovaiarelli@gmail.com (A.V.); laura.rienzi@ivirma.com (L.R.); ubaldifm@gmail.com (F.M.U.); 4 Gynecology and Obstetrics 1U, Physiopathology of Reproduction and IVF Unit, Department of Surgical Sciences, S. Anna Hospital, University of Turin, 10126 Turin, Italy; andrea88carosso@gmail.com; 5 Department of Biomolecular Sciences, University of Urbino “Carlo Bo”, 61029 Urbino, Italy; 6 Department of Pharmacy and Health Sciences and Nutrition (DFSSN), University of Calabria, 87036 Cosenza, Italy

**Keywords:** DuoStim, oocyte accumulation, follicular phase stimulation, oocyte vitrification, time to live birth

## Abstract

This study compared the DuoStim protocol with two conventional follicular phase stimulations for vitrified oocyte accumulation in poor-prognosis patients undergoing PGT-A. A retrospective analysis of 112 IVF cycles was conducted, with 66 cycles among patients undergoing DuoStim (DS-Group) and 46 among patients undergoing conventional follicular phase stimulations (DF-Group). The primary outcome was the time to live birth, while secondary outcomes included clinical pregnancy rate, miscarriage rate, live birth rate, and cumulative live birth rate. The final analysis included 66 patients in the DS-Group and 40 in the DF-Group, as 6 women (13%) in the DF-Group discontinued treatment after the first stimulation. Oocyte yield was similar between groups (8.4 ± 3.9 in DS-Group vs. 8.2 ± 4.0 in DF-Group, *p* = 0.80), as was the number of euploid blastocysts (0.9 ± 1.2 vs. 1.1 ± 1.1, *p* = 0.37). The cumulative live birth rate was 22.7% in the DS-Group and 25% in the DF-Group (multivariate odds ratio adjusted for maternal age and male factor: 1.05, *p* = 0.93). The time to live birth was significantly shorter in the DS-Group (81.5 ± 15.5 days) compared to the DF-Group (153.7 ± 78.2 days, *p* < 0.001). DuoStim showed similar efficacy but a shorter time to live birth.

## 1. Introduction

One of the major steps of IVF is ovarian stimulation (OS) with exogenous gonadotropin administration from the beginning of the follicular phase. This practice is based on the traditional theory of a ‘single recruitment episode’, stating that a single cohort of follicles starts growing in each ovarian cycle at the time of menstruation. In 2003, it was first reported that multiple waves of follicular recruitment occur in humans across a single ovarian cycle [1,2]. The evidence of multiple follicular growth waves and the advances in the field of oocyte and embryo cryopreservation allowed adopting novel “unconventional” OS protocols, characterized by asynchrony between the hormonal enhancement of follicular development and the endometrial cycle. These novel protocols start in different phases of the cycle: any day (random start), in the luteal phase (luteal phase stimulation), or even twice in the same ovarian cycle back-to-back, and may be used to increase oocyte yield in specific patient populations. Among new protocols, DuoStim allows the performance of a second round of OS immediately following the first oocyte pick-up (OPU), finally retrieving oocytes twice in less than one month [3,4]. A proof of concept in poor-prognosis patients with poor ovarian reserve and/or advance maternal age showed that fertilization, blastulation, and euploid blastocyst formation rates were comparable after the first and the second rounds of OS in the same menstrual cycle [5]. Interestingly, the cohort of oocytes obtained at the second OPU was on average one oocyte larger than after the first, and the oocytes had comparable competence, thus resulting in a higher number of blastocysts available for PGT-A [6]. In a subsequent multicentre study, euploid blastocysts obtained after the second OS showed the same clinical, obstetric, and perinatal outcomes as those derived from the first round, confirming that DuoStim is a feasible, safe, and efficient approach to increase the chance of obtaining a euploid blastocyst [7]. DuoStim was also shown as being the most promising approach in managing women with poor prognosis, such as those fulfilling the Bologna criteria [8] and patients for whom retrieving oocytes in a short timeframe is critical, such as oncologic patients seeking fertility preservation [9,10]. The DuoStim protocol was also applied to manage expected low/suboptimal responders by storing vitrified mature oocytes obtained after the first OS and then thawing and inseminating them together with fresh oocytes retrieved after the second OS [11]. The option of accumulating vitrified oocytes was supported by the effectiveness and safety of vitrification [12], which was proven to obtain success rates comparable to those of fresh oocytes [13,14]. Previous findings even showed that oocyte vitrification does not increase the aneuploidy rate in the blastocyst and that the availability of euploid embryos may be increased by using vitrified oocytes together with fresh ones [15]. Indeed, enlarging the cohort of oocytes available for fertilization would represent an intriguing approach to raising the likelihood of finding at least one euploid embryo. Obviously, this goal may be also reached by pooling vitrified eggs obtained after OS performed in different follicular phases within the same ovarian cycle. To this regard, recently, one randomized controlled trial showed inconclusive results comparing two consecutive stimulations (BiSTIM protocol) with two conventional OS cycles [16]. Differently, other authors suggested the application of DuoStim to shorten the time to obtain euploid blastocysts (TTEB) [17]. Our aim was to provide evidence on the efficacy and efficiency of DuoStim (in which mature oocytes are frozen after the first egg retrieval and used after the second retrieval) versus two conventional ovarian stimulations, a procedure which also involves the vitrification of oocytes after the first OPU in order to inseminate them together with fresh oocytes obtained from the second OPU. To this end, our primary endpoint was the time to live birth (TTLB).

## 2. Materials and Methods

### 2.1. Study Design and Patient Population

**Figure 1:**
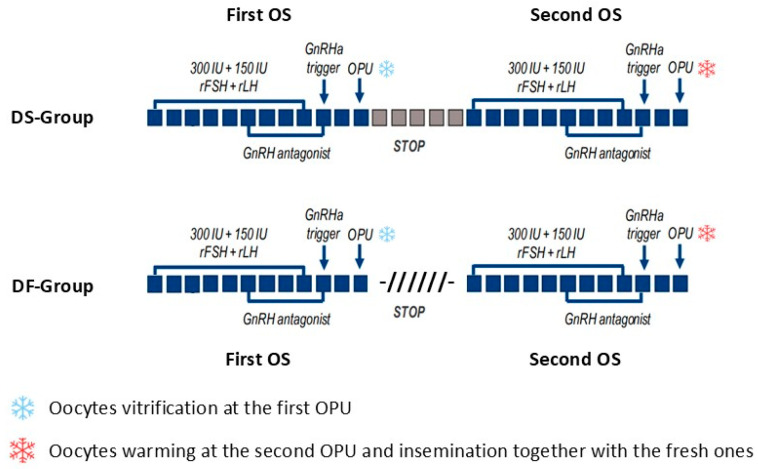
Ovarian stimulation (OS) strategies for the treatment of poor/suboptimal responders undergoing PGT-A. Patients were suggested to undergo either two consecutive OS sessions in the same ovarian cycle (DS-Group) or two conventional OS sessions started in the follicular phase of two ovarian cycles (DF-Group). In the DS-Group, five days after the first oocyte retrieval, luteal phase stimulation was performed with an identical protocol (each square represents a day of the cycle). In the DF-Group, a variable time period after the first oocyte retrieval, a second conventional OS started in the follicular phase of a new but not always consecutive cycle was performed with an identical protocol (slanting lines represent the time period between the first oocyte retrieval and the start of the second OS). In both groups, oocyte vitrification was applied at the first OPU followed by their warming and insemination together with the fresh ones retrieved at the second OPU.

This observational study included 112 patients, expected to be poor or suboptimal responders, who were candidates for PGT-A (mean age: 40.0 ± 2.6 years, range 33–45) at our private IVF clinic and who underwent 112 IVF cycles between March 2019 and March 2022. All patients fulfilled at least two of the following criteria: woman’s age ≥ 35 years, circulating anti-Mullerian hormone (AMH) ≤ 1.5 ng/mL, antral follicle count (AFC) ≤ 6, and/or ≤5 retrieved oocytes in a previous IVF session. As the two study populations were homogeneous, no preselection process or patient matching was performed. Ethics committee approval was obtained for the retrospective analysis of pseudonymized data. Extensive counselling was conducted by the same clinicians based on a woman’s age and the expected risk of embryonic aneuploidies, and was focused on the importance of retrieving the highest possible number of oocytes and embryos to increase the chance of obtaining at least one euploid blastocyst for transfer. Before the present study, we would accumulate vitrified oocytes obtained after conventional OS started in the follicular phase of two different menstrual cycles; with time, we gradually introduced DuoStim, encouraged by the emerging evidence in the literature. Starting in March 2019, two alternative vitrified oocyte accumulation strategies were proposed to patients with the same expenses: (i) two consecutive OS sessions in the same ovarian cycle, with oocyte vitrification after the first OPU, a second administration of exogenous gonadotropins 5 days after the OPU, and then warming of the oocytes and insemination together with fresh oocytes retrieved at the second OPU (DuoStim Group, DS-Group); or (ii) vitrified oocyte accumulation thought two conventional OS sessions started in the follicular phase of two different ovarian cycles when the ovaries showed a basal condition (non-functional cyst and/or residual corpus luteum). Based on this condition, the two stimulations were not always consecutive but took place within three months (27.7 ± 25.5 days, range 8–90 days, between the first OPU and the start of the second OS), with oocyte vitrification at the first OPU, followed by their warming and insemination together with the fresh gametes retrieved at the second OPU (Double-Follicular Group, DF-Group). Moreover, patients in DF-Group could decide to postpone the second OS over a variable period of time, depending either on their personal choice or when the ovaries returned to basal ultrasound condition (Figure 1). In both groups, the time between the two OPUs was calculated as the time between the day of the first OPU and the day of the second OPU. Among all women undergoing IVF in the study period, 112 met the inclusion criteria: 66 underwent DuoStim (DS-Group), and 46 underwent the conventional approach with two-follicular-phase OS in different ovarian cycles (DF-Group). Six women initially included in the DF-Group refused to undergo the second OS, either discontinuing the treatment or using only the oocytes that were vitrified after the first OPU (Supplementary Figure S1).

### 2.2. Ovarian Stimulation (OS), IVF and PGT-A

The first OS was started in both groups on day 2 of the menstrual cycle, with a starting dose of 300 IU/d rFSH+rLH at a 2:1 ratio (Pergoveris^®^, Merck, Darmstadt, Germany), that was chosen according to the woman’s age, AFC, and AMH. Circulating E2 and transvaginal US examination were performed every second day from stimulation day 6–7 to monitor follicular growth, adapting the gonadotropin dose when required. GnRH antagonist (cetrorelix, Cetrotide, Merck-Serono; ganirelix, Orgalutran, MSD, Rahway, NJ, USA) was administered daily (0.25 mg subcutaneously) from stimulation day 6. When at least two follicles reached 18 mm in mean diameter, with appropriate E2 levels, 0.2 mg triptorelin acetate (Decapeptyl, Ferring, Saint-Prex, Switzerland) was administered to trigger ovulation. US-guided oocyte pick-up (OPU) was performed 35–37 h later under local anaesthesia (paracervical block). Follicular fluids were aspirated and immediately observed under a stereomicroscope. Cumulus–oocyte complexes (COCs) were washed in buffered medium (Gamete medium, Vitrolife, Gothenburg, Sweden), and within 2 h of the OPU, oocytes and cumulus cells were separated by gently pipetting in 600 µL buffered medium containing hyaluronidase (HYASE-10X, Vitrolife, Sweden). Metaphase II (MII) oocytes were vitrified using the Kitazato protocol (Kitazato, Shizuoka, Japan). In the DS-Group, the second OS was started 5 days after the first OPU with the same protocol and same daily dose regardless the number of antral follicles counted at the scan [18], while in the DF-Group the second OS was started on day 2 of the following menstrual cycle only when there were basal ovarian conditions for starting a new stimulation. In both groups, the accumulated vitrified oocytes were warmed and pooled with the fresh ones retrieved at the second OPU and then were inseminated by intra-cytoplasmic sperm injection (ICSI) within 4 h.

Normal fertilization was confirmed when the presence of two pronuclei (2PN) and the extrusion of the second polar body were observed 16–18 h after ICSI. Zygotes were cultured in 700 µL of cleavage medium (Vitrolife, Gothenburg, Sweden) overlaid with 550 µL of mineral oil (LifeGuard Oil, LifeGlobal IVF, Trumbull, CT, USA) up to day 3 of development; at this stage, the culture medium was changed using 700 µL of a stage-specific medium (Sequential Blast, Origio, Ballerup, Ireland) up to the fully expanded blastocyst stage (day 5–7). Embryo culture was carried on in a controlled, humidified atmosphere (37 °C, 6% CO_2_) with low oxygen tension (5%) in BT37 (Planer, Origio, Ballerup, Ireland) benchtop incubators. Inseminated oocytes and embryos derived from the first and second OS sessions were cultured separately. Trophectoderm biopsy was performed on all viable blastocysts using laser-assisted zona opening and sequential fragment retrieval. Vitrification was performed within 30 min after trophectoderm biopsy on collapsed blastocysts with Cryotop carrier using a vitrification kit (Kitazato, Shizuoka, Japan). Comprehensive chromosome testing was conducted by next-generation sequencing (NGS) at an external lab. The method was designed to specifically identify constitutive whole-chromosome non-mosaic aneuploidies. In the presence of euploid blastocyst(s), a vitrified-warmed euploid single-embryo transfer (SET) was performed during the next menstrual cycle using a Sydney Guardia soft catheter (Cook, Bloomington, IL, USA) under transvaginal US guidance. Embryo transfer (ET) was performed after either hormone replacement therapy (HRT) (17.2%, N = 5/29 DS-Group; 36%, N = 9/25 DF-Group, *p* = 0.12) or on a natural cycle based on patient characteristics and a doctor’s decision. In HRT cycles, oral estradiol valerate (Progynova, Bayer, Leverkusen, Germany) was administered as follows: 2 mg twice a day for the first 4 days and three times a day for the rest of the therapy period. When the endometrial thickness reached at least 7–8 mm with a trilaminar (type 1) aspect, vaginal micronized progesterone was administrated at a dose of 600 mg/day (Progeffik 200 mg, Effik, Cinisello Balsamo, Milan, Italy). After 6 days of micronized progesterone, we planned ET. In the case of a natural cycle, ET was performed 7 days after an ovulation trigger or 6 days after the detection of the urinary LH peak. Progesterone was administered until the day of the pregnancy test or until week 9 in case of pregnancy. The pregnancy test was performed 11 days after SET. Clinical pregnancy was determined by ultrasound visualization of a gestational sac with foetal heartbeat at 5–7 weeks. The miscarriage rate was calculated as the number of pregnancy losses divided by the number of clinical pregnancies.

### 2.3. Statistical Analysis

The primary endpoint of the study was the time to live birth (TTLB), calculated as the time between the starting day of the first OS and the day of the ET resulting in a live birth. Power analysis was performed using the G*Power v3.1 software. Secondary outcomes were the number of retrieved oocytes, blastulation rate, euploid blastocyst rate per inseminated oocyte, percentage of patients with at least one euploid blastocyst, and cumulative live birth rate (CLBR) per cycle and per intention to treat (ITT). Among secondary outcomes, we also calculated other indicators: (a) the time to obtain at least one euploid blastocyst (TTEB), calculated as the time between the starting day of the first OS and the day on which one euploid blastocyst was obtained, and (b) the time to cycle termination (TTCT), calculated as the time between the starting day of the first OS and the day of a second OPU (in absence of MII oocytes obtained after both stimulations), of a pronuclear check (in case of no fertilized oocytes obtained after both stimulations), of the end of embryo culture (in case of no blastocyst obtained after both stimulation), of the last ET (in case of no live birth), or of the first ET resulting in a live birth. Analysis was conducted both comparing the two regimens (DS-Group versus DF-Group) and the first OS versus second OS for each protocol. After controlling the Gaussian distribution of the data with the Shapiro–Wilk test, the comparison among groups was performed using the GraphPad Prism V7 software, applying Student’s parametric *t*-test, the non-parametric Mann–Whitney test, or the Chi-square test as appropriate. Regression analysis was performed with SPSS V30 (IBM, Armonk, NY, USA). All factors were tested as potential confounders in order to adjust clinical outcomes. Continuous variables were expressed as mean ± standard deviation (SD), whereas categorical variables were expressed as absolute values and percentages. All statistical tests were two-sided, and a *p* value ≤ 0.05 was considered statistically significant.

## 3. Results

### 3.1. DuoStim and Two Conventional Stimulations Show Similar Efficacy, with Comparable Oocyte Yield, Euploid Blastocysts, and CLBR

The baseline clinical characteristics and the embryological outcomes of the two patient groups are shown in Table 1. No significant differences were observed regarding the demographic data or the variables related to OS; in particular, the total number of retrieved oocytes was comparable, as were the total number of available blastocysts and the euploid blastocyst rate. The only significant difference, as expected, was a shorter interval between the two OPUs in the DS-Group compared to the DF-Group (Table 1).

We observed comparable outcomes for positive pregnancy tests per first euploid blastocyst transfer (65.4%, N = 17/26 − CI 044–0.82 versus 72.2%, N = 13/18 − CI 0.46–0.89 in the DS-Group and DF-Group, respectively; *p* = 0.63), clinical pregnancy rate (57.7%, N = 15/26 − CI 0.37–0.76 versus 44.4%, N = 8/18 − CI 0.22–0.69, in the DS-Group and DF-Group, respectively; *p* = 0.39), miscarriage rate (13.3%, N = 2/15 − CI 0.02–0.42 versus 12.5%, N = 1/8 − CI 0.007–0.53 in the DS-Group and DF-Group, respectively; *p* = 0.95), and live birth rate per first euploid blastocyst transfer (50%, N = 13/26 − CI 0.30–0.70 versus 38.9%, N = 7/18 − CI 0.18–0.64, in the DS-Group and DF-Group, respectively; *p* = 0.47). The CLBR per cycle was 22.7%, N = 15/66 − CI 0.14–0.35 versus 25%, N = 10/40 − CI 0.13–0.42, in the DS-Group and DF-Group, respectively (*p* = 0.79; multivariate odds-ratio adjusted for maternal age and male factor: 1.05, 95%CI 0.37–2.93; *p* = 0.93), and was comparable even when calculated for ITT (22.7%, N = 15/66 − CI 0.14–0.35 versus 21.7%, N = 10/46 − CI 0.11–0.37 in the DS-Group and DF-Group, respectively; *p* = 0.90). Finally, we observed a similar proportion of patients with surplus euploid blastocysts after a live birth (35.7%, N = 5/14 − CI 0.14–0.64 versus 40%, N = 4/10 − CI 0.14–0.73 in the DS-Group and DF-Group, respectively; *p* = 0.83), with a mean number of 2.6 ± 1.1 available blastocysts in the DS-Group (range 2–5) versus 2.7 ± 0.5 in the DF-Group (range 2–3) (*p* = 0.85). Comparing the DS-Group (N = 66) with the DF-Group (N = 40), all cycles in both groups obtained at least one oocyte; 97% (N = 64/66) and 100% (N = 40/40) of cycles, respectively, had at least one MII oocyte (*p* = 0.27); 93.9% (N = 62/66) and 100% (N = 40/40) of cycles, respectively, had at least one 2PN-zygote (*p* = 0.11); 72.7% (N = 48/66) and 80% (N = 32/40) of cycles, respectively, had at least one blastocyst (*p* = 0.40); 39.4% (N = 26/66) and 47.5% (N = 19/40) of cycles, respectively, had at least one euploid blastocyst (*p* = 0.41); and finally, 22.7% (N = 15/66) and 25% (N = 10/40) of cycles, respectively, had at least one live birth (*p* = 0.65).

### 3.2. DuoStim Shows Better Efficiency, with a Shorter Time to Obtain Euploid Blastocysts, Achieve a Live Birth, and Conclude the Cycle

**Figure 2:**
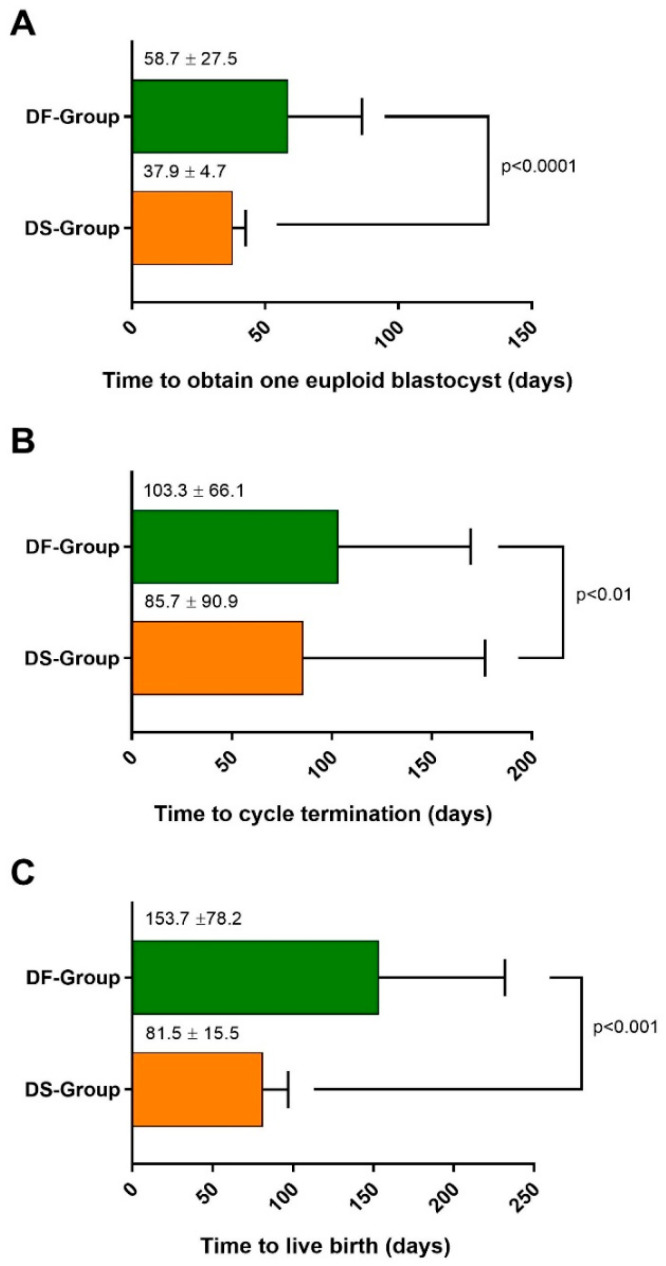
Timeline indicating the number of days needed (**A**) to obtain at least one euploid blastocyst, (**B**) to conclude the IVF cycle, and (**C**) to obtain an ongoing pregnancy (≥22 gestational weeks) in each of the two OS regimens. All times were significantly shorter using DuoStim.

The achievement of at least one euploid blastocyst (TTEB = time between the starting day of the first COS and the day in which one euploid blastocyst was obtained) required 37.9 ± 4.7 days in the DS-Group versus 58.7 ± 27.5 days in the DF-Group (*p* < 0.0001; Figure 2A). The time to cycle termination (TTCT, see Statistics for definition) was significantly reduced in the DS-Group (85.7 ± 90.9 versus 103.3 ± 66.1 days; *p* < 0.01; Figure 2B). Finally, the primary outcome of the study, the time to achieve a live birth (TTLB = time between the starting day of the first OS and the day of the ET resulting in a live birth) was significantly shorter in the DS-Group (81.5 ± 15.5 versus 153.7 ± 78.2 days; *p* < 0.001; Figure 2C).

## 4. Discussion

Treatment personalization with tailored therapeutic strategies has a pivotal role in modern IVF, aiming at improving both efficacy and efficiency. Laboratory add-ons, such as intra-cytoplasmatic sperm injection (ICSI), vitrification, blastocyst culture, and comprehensive chromosome testing (PGT-A), may all contribute to improve outcomes in specific patients [19]. A tailored OS is critical as well: the POSEIDON group defined as successful an OS regimen that “allows retrieving the number of oocytes needed to obtain at least one euploid blastocyst to transfer” [20]. Recently, the evidence that competent MII oocytes can be obtained while also administering gonadotropins in the luteal phase has further improved the options to personalize OS [21]. Notably, performing a second OS round in the same ovarian cycle provides the novel opportunity to increase the cohort of oocytes available for insemination and the chance of obtaining at least one euploid blastocyst in a short timeframe via a multi-cycle approach [22]. Indeed, two complementary systematic reviews and meta-analyses demonstrated that DuoStim can offer outcomes similar to those of two conventional OS cycles performed in the follicular phase, but in a shorter timeframe [23,24,25]. Herein we compared DuoStim versus two conventional OS cycles performed in the follicular phase of two different ovarian cycles combined with vitrified oocytes after the first stimulation in both groups as strategies to maximize the number of oocytes available for insemination and the number of blastocysts for PGT-A. Both regimens resulted comparably in obtaining an average of approximately eight oocytes and two blastocysts, which can be considered a fairly good result for poor/suboptimal responders. Similarly, in both groups, the blastocysts derived from vitrified oocytes showed a comparable euploidy rate compared to those deriving from fresh eggs, in agreement with previous studies [26,27]. Interestingly, a comparable proportion of patients with surplus euploid blastocysts, suitable for multiple warming cycles, were observed in the two groups. To this regard, long-term cryo-storage of human embryos is useful to increase the CLBR and is safe, as recently supported by a systematic review and meta-analysis that provided reassuring evidence [28]. Overall, in the present study DuoStim had comparable efficacy to two-follicular-phase OS in terms of embryological outcomes as well as clinical pregnancy rate, miscarriage rate, live birth rate, and CLBR. However, time indicators were all significantly improved by DuoStim: in fact, the time necessary to obtain at least one euploid blastocyst, to achieve a live birth (our primary outcome), or to conclude the cycle were all significantly, although moderately, shorter when DuoStim was used. These findings are important for counselling because shortening times is priceless for women of advanced maternal age, with poor ovarian reserves, or with multiple previously failed IVF attempts. In addition, the level of patient compliance with the user-friendly DuoStim ovarian stimulation protocol was very high in our clinical context. Interestingly, we observed a significantly higher drop-out rate among patients in the DF-Group arm, where 13% of the patients did not return within one year for the second OS. These patients underwent two conventional ovarian stimulations, starting in the follicular phase of two different, though not always consecutive, cycles. They chose to postpone the second OS for a variable period, based on emotional stress or lack of basal condition of the ovaries [29,30]. We cannot establish with certainty the reasons why these couples dropped out, but it may be remarked that DuoStim has been shown to consistently reduce treatment discontinuations in poor responders fulfilling the Bologna criteria [8] and shorten the time to euploid blastocyst retrieval [6], as providing a particularly intensive and rapid stimulation protocol may encourage patients to carry on with their treatment schedule. In terms of cost-effectiveness, DuoStim resulted in lower costs per “patient with at least one LB” in our previous reports [31]. Based on these data, DuoStim can be considered a “one-and-done” strategy (i.e., the completion of an average-sized family [≥2 LBs] after a single complete IVF cycle) whose cost is reasonably higher than the conventional approach.

Regarding potential long-term hormonal disruptions, current evidence does not suggest any significant long-term adverse effects resulting from closely timed stimulations. Although DuoStim involves two consecutive stimulations in one ovarian cycle, no increased incidence of hormonal imbalances or related complications have been reported in the available literature. Nevertheless, long-term follow-up data are still limited, and further research is encouraged to fully confirm the long-term safety profile.

Finally, in our clinical practice, the amount of bleeding experienced by patients undergoing the DuoStim protocol after oocyte pick-up (OPU) was comparable to that experienced by patients undergoing conventional in vitro fertilisation (IVF) procedures. The procedures’ duration and the timing of oocyte collection, insemination, and embryo culture were also similar. Previously, gestational and perinatal outcomes (including gestational weeks, gestational diabetes, placental pathologies, preterm delivery rate, low birth weight, being small or large for gestational age, and neonatal malformations) were assessed according to the World Health Organization [7]. The results were similar to those of conventional OS protocols. Overall, patients undergoing the DuoStim protocol are not at a higher risk of surgical or pregnancy complications.

DuoStim could offer a valuable opportunity for oncology patients undergoing oocyte vitrification for fertility preservation, as they are often required to minimize delays in order to begin cancer treatment promptly. In this context, the DuoStim protocol could significantly shorten the time required to collect an adequate number of oocytes. By accumulating vitrified oocytes or embryos through multiple conventional stimulations or DuoStim cycles, it becomes possible to increase the number of viable embryos from separate oocyte batches, overcoming the limitations of a single-cycle strategy. Specifically, although DuoStim would also comply with the “one-and-done approach” [32] and offers patients the opportunity to achieve multiple blastocyst formations in the shortest possible time, viewing the treatment plan through the lens of family planning could be particularly important for patients of advanced maternal age and/or diminished ovarian reserve. In such cases, a shift from a cycle-to-cycle approach to a multi-cycle approach could be beneficial [33].

## 5. Conclusions

Although observed in a limited sample size of poor-prognosis patients undergoing vitrified oocyte accumulation for PGT-A, the DuoStim protocol was found to be as efficient as performing two stimulations in the follicular phases of two different ovarian cycles in terms of live birth rate and CLBR. However, DuoStim shows better efficiency, as it leads to a significantly shorter time to obtain at least one euploid blastocyst, achieve a live birth, and/or conclude the IVF treatment.

## Figures and Tables

**Table 1:** Clinical and embryological characteristics of 66 patients undergoing the DuoStim protocol (DS-Group) versus 40 undergoing the double-follicular-stimulation protocol (DF-Group). BMI = Body Mass Index; FSH = Follicle-Stimulating Hormone; LH = Luteinizing Hormone; AMH = Anti-Müllerian Hormone; OS = Ovarian Stimulation; OPU = Ooctyte Pick-Up; MII = Metaphase II; 2PN = 2 Pronuclei.

	DS-Group (n = 66)	DF-Group (n = 40)	*p*-Value
Woman’s age (years)	39.9 ± 2.5	40.1 ± 2.7	0.71
Partner’s age (years)	41.7 ± 4.9	42.6 ± 5.8	0.48
Previous IVF cycles (n)	0.7 ± 1.2	1.0 ± 1.6	0.29
BMI (kg/m^2^)	21.9 ± 3.5	22.1 ± 3.5	0.73
Basal FSH (IU/I)	9.9 ± 3.4	10.3 ± 3.0	0.18
Basal LH (IU/I)	5.1 ± 1.9	5.1 ± 2.0	0.99
AMH (ng/mL)	1.1 ± 1.3	0.9 ± 0.7	0.72
Antral Follicle Count (n)	9.7 ± 5.6	8.7 ± 4.0	0.47
Duration of OS (days, range)	First: 10.4 ± 2.3 (5–17) Second: 12.1 ± 2.5 (6–18)	First: 11.1 ± 2.6 (7–19) Second: 11.6 ± 2.5 (7–17)	0.31 0.42
Time between first and second OPU (days, range)	18.6 ± 2.8 (12–25)	40.9 ± 25.5 (17–103)	<0.0001
Time between the first OPU and the start of the second OS (days, range)	5 (-)	27.7 ± 25.5 (8–90)	<0.0001
Retrieved oocytes (n)	8.4 ± 3.9	8.2 ± 4.0	0.80
Oocyte survival rate after vitrification/warming (%)	87.6 ± 27.3%	87.3 ± 29.8%	0.65
MII oocytes after two OS (n)	5.9 ± 3.3	5.7 ± 3.1	0.65
2PN zygotes (n)	4.9 ± 2.7	5.0 ± 2.9	0.97
Blastocysts (n)	2.1 ± 1.8	2.3 ± 2.1	0.82
Euploid Blastocysts (n)	0.9 ± 1.2	1.1 ± 1.1	0.37
Euploid blastocysts/oocyte (%)	9.8 ± 11.9%	12.3 ± 12.6%	0.32
Euploid blastocysts/MII oocyte (%)	12.8 ± 14.6%	16.3 ± 15.2%	0.23
Euploid blastocysts/2PN zygote (%)	15.9 ± 18.7%	17.3 ± 15.9%	0.37
Euploid blastocysts/biopsied (%)	28.9 ± 32.4%	34.1 ± 34.5%	0.52

## Data Availability

The data presented in this study are available on request from the corresponding author.

## Supplementary Materials

The following supporting information can be downloaded at: https://www.mdpi.com/article/10.3390/life15060899/s1, Figure S1: Patient flow chart.

## Abbreviations

The following abbreviations are used in this manuscript:

BMI	Body Mass Index
FSH	Follicle-Stimulating Hormone
LH	Luteinizing Hormone
AMH	Anti-Müllerian Hormone
MII	Metaphase II
2PN	2 Pronuclei
OS	Ovarian Stimulation
IVF	In Vitro Fertilization
OPU	Oocyte Pick-up
ET	Embryo Transfer
ITT	Intention To Treat
CLBR	Cumulative Live Birth Rate
PGT-A	Preimplantation Genetic Test for Aneuploidies
TTEB	Time To Obtain One Euploid Blastocyst
TTLB	Time To Live Birth
TTCT	Time To Cycle Termination
