# Supplementary material for: DuoStim Shows Comparable Efficacy but Better Efficiency than Two Conventional Stimulations in Poor/Suboptimal Responders Undergoing Vitrified Oocyte Accumulation for PGT-A
Source: Life (Basel). 2025 May 31;15(6):899. doi: 10.3390/life15060899 (PMC12193753; doi:10.3390/life15060899)
Supplement: Supplementary file 1 [file life-15-00899-s001.zip › life-3605273-supplementary.pdf]

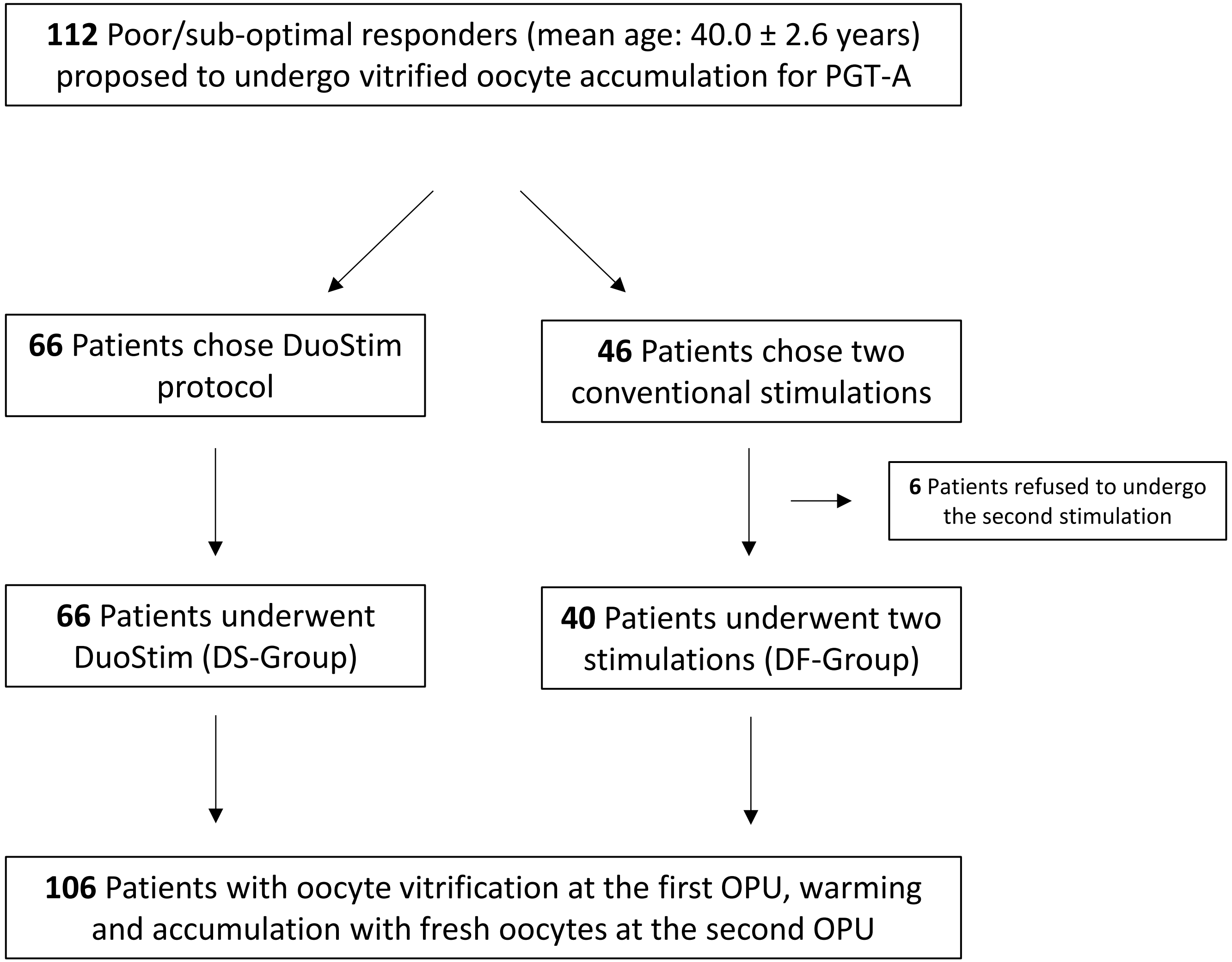
112 Poor/sub-optimal responders (mean age: 40.0 ± 2.6 years) proposed to undergo vitrified oocyte accumulation for PGT-A
66 Patients chose DuoStim protocol
46 Patients chose two conventional stimulations
6 Patients refused to undergo the second stimulation
66 Patients underwent DuoStim (DS-Group)
40 Patients underwent two stimulations (DF-Group)
106 Patients with oocyte vitrification at the first OPU, warming and accumulation with fresh oocytes at the second OPU
